# Achieving malaria testing and treatment targets for children under five in Mozambique: a cost-effectiveness analysis

**DOI:** 10.1186/s12936-022-04354-9

**Published:** 2022-11-07

**Authors:** Anton L. V. Avanceña, Angie Miller, Neide Canana, Janeth Dula, Abuchahama Saifodine, Baltazar Cadrinho, Elisa M. Maffioli

**Affiliations:** 1 Department of Health Management and Policy, School of Public Health, University of Michigan, Ann Arbor, MI USA; 2 Malaria Consortium, Maputo, Mozambique; 3 Health Policy and Systems Programme, Instituto Nacional de Saude, Maputo, Mozambique; 4 United States President’s Malaria Initiative, USAID, Maputo, Mozambique; 5 National Malaria Control Programme, Ministry of Health, Maputo, Mozambique; 6 Present Address: Health Outcomes Division, College of Pharmacy, University of Texas at Austin, Austin, TX USA

**Keywords:** Cost-effectiveness, Malaria, Testing, Treatment, Rapid diagnostic testing, Artemisinin combination therapy

## Abstract

**Background:**

The entire population of Mozambique is at risk for malaria, which remains one of the leading causes of death. The 2017–2022 National Malaria Strategic Plan focuses on reducing malaria morbidity and mortality in high- and low-transmission areas. This study aimed to estimate the costs and health benefits of six variations of the World Health Organization’s “test-and-treat” strategy among children under five.

**Methods:**

A decision tree model was developed that estimates the costs and health outcomes for children under five. Data on probabilities, costs, weights for disability-adjusted life years (DALYs), and quality-adjusted life years (QALYs) were based on peer-reviewed, grey literature, and primary data analysis of the 2018 Malaria Indicator Survey. Six scenarios were compared to the status quo and calculated the incremental cost-effectiveness ratio (ICER) in terms of cost per QALY gained, DALY averted, and life saved. Deterministic and probabilistic sensitivity analyses were conducted to understand the effect of parameter uncertainty on the findings.

**Results:**

In the base case, reaching the target of 100% testing with rapid diagnostic tests (RDTs; Scenario 1) is more cost-effective than improving the testing rate alone by 10% (Scenario 2). Achieving a 100% (Scenario 3) or a 10% increase in treatment rate (Scenario 4) have ICERs that are lower than Scenarios 1 and 2. Both Scenarios 5 and 6, which represent combinations of Scenarios 1–4, have lower ICERs than their constituent strategies on their own, which suggests that improvements in treatment are more cost-effective than improvements in testing alone. These results held when DALYs averted or lives saved were used as health outcomes. Deterministic and probabilistic sensitivity analyses revealed that the cost-effectiveness of Scenarios 1–6 are subject sensitive to parameter uncertainty, though Scenarios 4 and 5 are the optimal choice when DALYs averted or QALYs gained were used as the measure of health outcomes across all cost-effectiveness thresholds.

**Conclusions:**

Improving testing rates alone among children at risk for malaria has the potential to improve health but may not be the most efficient use of limited resources. Instead, small or large improvements in treatment, whether alone or in conjunction with improvements in testing, are the most cost-effective strategies for children under five in Mozambique.

**Supplementary Information:**

The online version contains supplementary material available at 10.1186/s12936-022-04354-9.

## Background

Despite the success of malaria prevention and treatment programmes, which have averted 1.5 billion cases and saved 7.6 million lives over the last two decades [1], nearly half the world's population still lives in areas at risk of malaria transmission. In 2020, malaria caused an estimated 241 million clinical episodes in 85 malaria-endemic countries, with the majority (95%) being in Africa [2]. The economic impact of malaria is estimated to cost the continent $12 billion every year, causing a “growth penalty” up to 1.3% per year in some countries [3, 4]. Among at-risk countries, Mozambique has the fourth-highest number of malaria cases in the world [2, 5] and still accounts for 30% of all deaths and 42% of deaths in children under 5 years old [6, 7]. Those affected, especially children and women [8, 9], and their families ultimately bear a large share of the burden through out-of-pocket payments of medical care and lost income due to time off work [10].

Even though awareness about malaria is widespread, appropriate management of malaria cases is far from universal in Mozambique due to the fragility of the health system: about half of malaria cases do not receive the recommended treatment while a large proportion of suspected cases that are test-negative are given anti-malarials [11, 12]. Presumptive diagnosis and treatment of malaria were for a long time the standard practice in sub-Saharan Africa, which lead to overtreatment with anti-malarials and contributed to the rapid emergence of drug-resistant strains [13, 14]. In 2010, the World Health Organization (WHO) changed the recommendation for the management of uncomplicated malaria from presumptive diagnosis to a “test-and-treat” strategy, or prescription of artemisinin-based combination therapy (ACT) after laboratory confirmation, either by microscopy or rapid diagnostic tests (RDTs). Most African countries, including Mozambique, adopted this recommendation. Mozambique’s 2017–2022 National Malaria Strategic Plan detailed the goal of the National Malaria Control Programme of the Ministry of Health (MoH) to reduce malaria morbidity and mortality by at least 40% [6], and the objective to “test 100% of suspected malaria cases and treat 100% of confirmed malaria cases at health facility and community level by 2022” was included among the six main objectives. To this end, the US President's Malaria Initiative and The Global Fund to Fight AIDS, TB, and Malaria (Global Fund) have allocated over $67.6 million for malaria case management in 2017–2020 [6]. Yet, despite the widespread availability of RDTs [15], adherence to WHO 2011 guidelines for malaria treatment remains a challenge in Mozambique and other sub-Saharan African countries [16].

Even if a lot is known about the morbidity and mortality associated with malaria in Mozambique [2], there is limited knowledge regarding the cost-effectiveness of existing malaria control policies carried out by the MoH and its partners [17], especially of malaria case management programs needed to reach the targets for 2022 [18]. This study implements a cost-effectiveness analysis (CEA) of alternative policy options of the WHO-recommended “test-and-treat” strategy compared to a status quo scenario [6]. This study relies on a decision-analytic model to estimate the costs of each scenario and their associated benefits in terms of quality-adjusted life years (QALYs) gained, disability-adjusted life years (DALYs) averted, and lives saved.

## Methods

### Overview

**Fig. 1:**
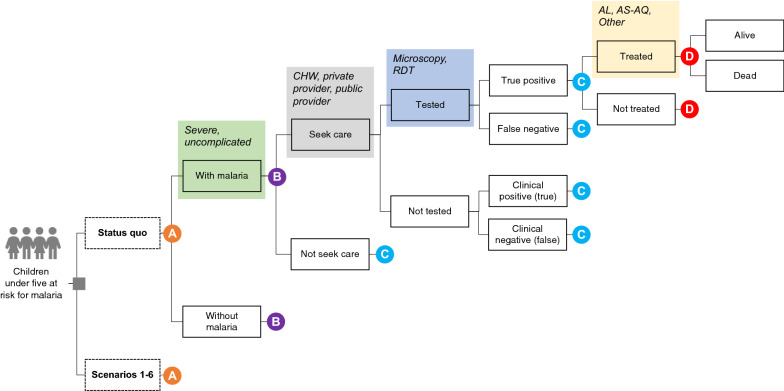
Schematic of decision tree model. A decision tree visually shows events and/or states of nature of different decisions or scenarios over a fixed time horizon. The grey square at the base of the tree is a decision node that shows the competing alternatives that a decision-maker is choosing between. Rectangles represent different events or states along the pathway of a decision; events from each divergence point in the tree are mutually exclusive and collectively exhaustive (e.g., “with malaria” and “without malaria”). Shaded squares represent additional branches in the tree that were omitted for simplicity. Each branch is associated with a probability. Some branches and states have been grouped, truncated, and represented by a coloured and labelled circle. CHW, community health worker. RDT, rapid diagnostic test

This CEA followed guidelines from the 2nd Panel on Cost-effectiveness in Health and Medicine [19] and the Consolidated Health Economic Evaluation Reporting Standards [20]. Using a decision tree model (Fig. 1), the costs and health benefits of six malaria testing and treatment scenarios (Table 1) were estimated in Mozambique compared to a status quo scenario. Data on probabilities, costs, and health outcome weights for QALYs and DALYs are based on peer-reviewed and grey literature identified through reviews conducted between June and December 2020, and primary data analysis of the 2018 Malaria Indicator Survey (MIS; Table 2). A limited health care sector perspective was used in the analysis, which means that only healthcare costs borne by payers (e.g. MoH, donor agencies) were included. The time horizon is one year, which precludes the need to discount costs and benefits.

**Table 1:** Description of modeled scenarios

Scenario	Description
Status quo	No changes to the current situation
1: 100% testing rate	Full compliance with national testing guidelines, which includes testing of all febrile or suspected cases with RDTs (i.e., phase-out of microscopy)
2: Increase testing rate by 10%	Increase overall testing rate by 10% (from 47 to 52%) and the RDT testing rate by 10% (from 69 baseline to 76%) across all types of providers^*^
3: 100% treatment rate	Full compliance with national treatment guidelines, which includes 100% treatment of all test-positive cases and treatment of uncomplicated cases with AL and severe cases with IV/IM artesunate
4: Increase treatment rate by 10%	Increase overall treatment rate by 10% (from 99 to 100%) and increase ACT treatment rate by 10% (from 84 to 92%)^*^
5: Scenarios 1 and 3	Combination of Scenarios 1 and 3; full compliance with testing and treatment guidelines, 100% testing with RDTs, and 100% treatment with AL for uncomplicated cases and IV/IM AS for severe cases
6: Scenarios 2 and 4	Combination of Scenarios 2 and 4; increase in overall testing and treatment rates by 10%, RDT testing rate by 10%, and ACT treatment rate by 10%

^*^The increase in testing and treatment rates can be a function of both supply and demand factors, including stockouts, staff training, and quality assurance and improvement activities

AL, artemether-lumefantrine; AS, artesunate; IM, intramuscular; IV, intravenous; RDT, rapid diagnostic test

**Table 2:** Summary of selected parameter inputs and assumptions

Input	Base value	Range	Distribution in PSA	Source
*Probabilities*				
Prevalence of malaria among children (6–59 months)	0.39	0.01–0.57	Beta	[21]
Uncomplicated case given malaria infection	0.87	0.65–0.9	Beta	[22]
Uncomplicated cases that seek care	0.69	0.86–0.93	Beta	[21]
Uncomplicated cases that seek care from CHWs given care-seeking	0.029	0.017–0.028	Beta	[21]
Uncomplicated cases that seek care from private providers given care-seeking	0.0062	0.0053–0.0089	Beta	[21]
Uncomplicated cases that are tested	0.47	0.65–0.74	Beta	[21]
Uncomplicated cases that are tested with RDTs by CHWs given testing	0.69	0.52–0.86	Beta	[11]
Uncomplicated cases that are tested with RDTs by private providers given testing	0.69	0.52–0.86	Beta	[11]
Uncomplicated cases that are tested with RDTs by public providers given testing	0.69	0.52–0.86	Beta	[11]
Microscopy sensitivity	0.96	0.87–1	Beta	[23]
Microscopy specificity	0.25	0.2–0.3	Beta	[23]
Uncomplicated cases that are treated given positive test result	0.99	0.74–1	Beta	[21]
Uncomplicated cases that are treated given negative test result	0.92	0.71–1	Beta	[21]
Uncomplicated cases that self-treat given no care-seeking	0.70	0.53–0.88	Beta	[24]
Uncomplicated cases that are treated with AL given a positive result	0.84	0.66–1	Beta	[21]
Uncomplicated cases that are treated with AL given a negative result	0.05	0.66–1	Beta	[21]
Uncomplicated cases that do not seek care that self-treat with AL	0.36	0.66–1	Beta	[21]
Treatment effectiveness of AL on uncomplicated cases	0.96	0.93–0.98	Beta	[25]
Treatment effectiveness of other drugs on uncomplicated cases	0	NA	NA	^*^
RDT sensitivity	0.90	0.83–0.9	Beta	[26]
RDT specificity	0.97	0.91–0.97	Beta	[26]
Death after untreated uncomplicated malaria	0.01	0.0075–0.0125	Beta	[27]
Accurate clinical diagnosis by CHWs	0.96	0.83–0.99	Beta	[28, 29]
Accurate clinical diagnosis by private providers	0.85	0.55–0.99	Beta	[24]
Accurate clinical diagnosis by public providers	0.76	0.54–1	Beta	[24]
Mortality from other causes	0.007	0.004–0.009	Beta	[8]
*Costs (in 2019 US$)*				
AL for uncomplicated malaria	1.68	1.68–1.68	Gamma	[30, 31]
AS/AQ for uncomplicated malaria	0.57	0.57–0.57	Gamma	[30, 31]
Other drugs taken by uncomplicated cases	0.29	0.29–0.36	Gamma	[30, 31]
AS for severe malaria	8.05	8.05–8.05	Gamma	[30, 31]
Microscopy	1.26	0.94–1.57	Gamma	[32]
RDT	1.54	1.15–1.92	Gamma	[33]
Clinical diagnosis/service delivery	4.18	4.16–4.19	Gamma	[9]
*Health outcome weights*				
Disability weight for uncomplicated malaria	0.006	0.002–0.012	Beta	[34]
Disability weight for severe malaria	0.133	0.088–0.19	Beta	[34]
Health utility for uncomplicated malaria	0.9802	0.9–1	Beta	[35]
Health utility for severe malaria	0.5363	0.41–0.66	Beta	[35]

For brevity, only probabilities that apply to uncomplicated malaria cases are shown here. For other probabilities used in the model, see Additional file 1

AL, artemether-lumefantrine; AS, artesunate; AS/AQ, artesunate-amodiaquine; CHWs, community health worker (also known as Agentes Polivalentes Elementares or APEs in Mozambique); RDT, rapid diagnostic test

^*^Value assumed or set by authors

^†^Base case value and/or range calculated by authors based on the cited references

### Model

A decision tree model was developed that estimates the costs and health outcomes for children under five living in Mozambique (Fig. 1). Decision tree models, which are one of many decision-analytic models that can be used in CEAs, visually track clinical pathways, events, and/or states of nature that individuals or populations experience over a fixed time horizon. Because of their simplicity, decision trees are best suited for modelling interventions where the time horizon is short and fixed [36]. The decision tree model was informed by previous models published in the literature [37, 38] and reviewed by co-authors and other staff at the MoH. The model was programmed in Microsoft Excel (Microsoft Corp, Redmond, WA).

### Data and sources

#### Transition probabilities

Each branch in the decision tree model is associated with an annual transition probability, which is listed in Table 2 and Additional file 1: Table S1. Probabilities specific to children under five were used where available. The prevalence of malaria in children is based on the 2018 MIS in Mozambique [21]. The distribution of malaria cases by severity is from a retrospective analysis of paediatric cases admitted to a rural hospital [22]. The probability of seeking care for suspected malaria and the probability of being tested is based on a cross-sectional study of female household heads in the Zambézia Province [39]. The probability of seeking care from different providers (i.e., public providers, private providers, and community health workers [CHWs]) was estimated using MIS data.

The probability of being tested with an RDT by type of provider is estimated using a cross-sectional study of various health facilities across three provinces in Mozambique [11]. The results reported in that study are similar to those in other studies [40]. The probability of testing with microscopy was assumed to complement RDT testing.

The sensitivity and specificity of RDTs and microscopy [23, 26] and the accuracy of clinical diagnosis by provider type [24, 28, 29] are based on studies done in and outside of Mozambique. The effectiveness of AL and AS on uncomplicated and severe malaria, respectively, are based on clinical trials in Mozambique [25, 41]. For untreated uncomplicated and severe malaria, the risk of death is based on published estimates [27, 42]. Death from other causes than malaria was estimated by subtracting the malaria-related death rate from the all-cause mortality rate among children under five in Mozambique, as reported in the Global Burden of Diseases Study (GBD) [8].

The 2018 MIS was analysed to estimate several probabilities, including the proportion of children under five who seek care from private or public providers for testing or treatment; the proportion of those who test positive for malaria through any test type; the proportion of those who test negative for malaria but still get treated; the proportion of those who seek care from CHWs or private providers when they have severe symptoms; the proportion of those who get tested using RDTs or microscopy when they have malaria symptoms; the proportion of those tested who get treated; the proportion of those who do not seek care, but get treated on their own; the proportion of those with severe malaria who use other treatment than intravenous or intramuscular (IV/IM) artesunate. Since the data do not distinguish between uncomplicated and severe malaria, this study assumed similar proportions across the two types, except for children with malaria symptoms being tested with any test type in 100% of the cases.

#### Costs

Anti-malarial drug costs were estimated using Global Fund price data and dosing recommendations from WHO treatment guidelines. The costs of microscopy, RDTs, and service delivery were taken from published studies in Mozambique [9, 32, 33]. Indirect costs such as lost income or caregiver costs were excluded from the analysis. All costs were inflated to 2019 US$.

#### Health benefits

The health benefits estimated in the decision tree model are QALYs gained, DALYs averted, and lives saved (i.e., deaths averted) for each modelled scenario.

QALYs represent a year that a person is alive weighted by that person's health‐related quality of life [43]. Preference-based health utilities are used to calculate QALYs for health states between perfect health and death; for this study, health utility estimates from a previous study that elicited preferences via a visual analog scale were used [35]. On the other hand, DALYs represent years of life lost due to premature death and disability and are the most commonly used measure of treatment effectiveness in economic evaluations in low- and middle-income countries [44]. To estimate years of life lost due to disability, disability weights from the most recent GBD were used [34]. Both QALYs and DALYs fall on a 0 to 1 scale and are preferred measures of health benefit in CEAs because they allow us to compare the efficiency of different interventions across diseases and conditions.

#### Cost-effectiveness analysis

The summary metric in CEAs is the incremental cost-effectiveness ratio (ICER). The ICER expresses how much each additional health gain is likely to cost. ICERs were calculated by dividing the incremental costs of a scenario by its incremental health benefits in terms of QALYs gained, DALYs averted, and lives saved.

$$ICER=\frac{Change in Costs}{Change in Effectiveness}=\frac{{C}_{Scenario}-{C}_{Status quo}}{{E}_{Scenario}-{E}_{Status quo}}$$

To interpret ICERs, they are often compared to context-specific cost-effectiveness thresholds. These thresholds are used as a convenient decision rule to estimate the opportunity costs of interventions and to determine whether health interventions are economically worthwhile investments or not [45, 46]. In this CEA, a range of cost-effectiveness thresholds were used that are based on recommended methods for low- and middle-income countries; this methodology departs from the now-withdrawn guidance from the WHO that suggested using 100% of gross domestic product (GDP) per capita as the cost-effectiveness threshold [47–50]. For Mozambique in 2019, the cost-effectiveness threshold range was $108–233 per QALY gained or 21–46% of the GDP per capita.

#### Sensitivity analysis

To explore the impact of parameter or second-order uncertainty on the study’s findings, two types of sensitivity analyses were conducted. The first is one-way or deterministic sensitivity analysis, where each transition probability, cost input, and utility value is varied one at a time from its lowest to highest value while keeping other parameters at their base value. Where available, low and high values were based on ranges in the literature; for some parameters, the authors determined reasonable bounds (Table 2 and Additional file 1: Table S1).

The second type of sensitivity analysis is probability sensitivity analysis (PSA), where parameters are varied simultaneously 10,000 times. PSA requires that each parameter included in the simulations is assigned a probability distribution with its corresponding parameters; transition probabilities and disability weights were assigned beta distributions, while cost inputs will be assigned a gamma distribution. Using the results of the PSA, average ICERs were calculated and produced cost-effectiveness acceptability curves, which graphically show which alternative is the optimal choice over a range of cost-effectiveness thresholds. The optimal choice was defined as the intervention with the highest net monetary benefit, which is the calculated by multiplying the benefit (either QALYs gained or DALYs averted) by a cost-effectiveness threshold and subtracting from the product the costs of the scenario [19]. An intervention with a positive net monetary benefit is considered economically efficient.

## Results

### Base case

Table 3 summarizes the base-case results for reaching testing and treatment targets in Mozambique. The analysis considers health benefits measured in QALYs gained and costs measured from a healthcare payer perspective (Additional file 1: Tables S2 and S3 shows the results of DALYs averted and lives saved). The results presented include incremental costs and benefits for each scenario (1 through 6) compared to the status quo, as well as their respective ICERs.

**Table 3:** Summary of base-case results

	Healthcare payer costs	QALYs gained	Incremental costs	Incremental QALYs gained	ICER (Cost per QALY gained)
Status quo	1.69	0.9587	NA	NA	NA
Scenario 1: 100% testing rate	2.06	0.9588	0.37	0.00011	3535
Scenario 2: Increase testing rate by 10%	1.72	0.9587	0.03	0.00001	3710
Scenario 3: 100% treatment rate	2.03	0.9590	0.34	0.00032	1085
Scenario 4: Increase treatment rate by 10%	1.75	0.9589	0.06	0.00018	325
Scenario 5: 1 and 3	2.61	0.9592	0.92	0.00045	2073
Scenario 6: 2 and 4	1.77	0.9589	0.09	0.00019	464

Incremental results are compared to the status quo scenario

ICER, incremental cost-effectiveness ratio; NA, not applicable; QALY, quality-adjusted life-year

Increasing the testing rate by 100% (Scenario 1) is more cost-effective than reaching a 10% testing rate (Scenario 2), as evidenced by the lower positive ICER. The difference in ICERs between these scenarios is small because the difference in the overall testing and RDT testing rates is also minimal (Table 1).

The ICERs for Scenarios 3 and 4 are $1,085 and $325 per QALY gained respectively (or $1,070 and $321 per DALY averted, respectively), which are lower than the ICERs for Scenarios 1 and 2. Thus, the model suggests that improvements in treatment alone are more efficient than improvements in testing alone. The model also found that eliminating presumptive treatment of test-negative *uncomplicated* cases causes the ICER of Scenarios 3 and 4 to shift minimally to $1,103 and $281 per QALY gained, respectively. On the other hand, presumptive treatment of test-negative *severe* cases can only be reduced from 100 to 89% in Scenarios 3 and 4 without negatively affecting the total health benefits. These results collectively indicate that presumptive treatment of test-negative cases can be potentially discontinued following improvements in overall treatment rates and treatment with ACT among children.

Scenarios 5 and 6, which are different combinations of Scenarios 1–4, have base-case ICERs that are also lower than Scenarios 1 and 2 but higher than Scenarios 3 and 4. Scenario 5, which combines Scenarios 1 and 3, is associated with an ICER of $2,073 per QALY gained (or $2,045 per DALY averted), which is twice the ICER of Scenario 3 and nearly half of Scenario 1. Scenario 6, which combines Scenarios 2 and 4, is associated with an ICER of $464 per QALY gained ($457 per DALY averted), which is the second-lowest after Scenario 4. These results suggest that concurrent improvements in testing and treatment, however small, are efficient strategies among children.

These results are robust to using alternative measures of health benefits, such as DALYs and lives saved (Additional file 1: Tables S2 and S3).

### Sensitivity analysis

The results of the one-way sensitivity analyses, which are shown in Additional file 1: Figs. S1–S6, reveal that extreme values of specific parameters can shift the efficiency of the modelled scenarios. For example, low malaria prevalence and low RDT sensitivity increase the ICER of Scenarios 1 and 2 (Additional file 1: Figs. S1 and S2, respectively). Increasing the treatment rate of uncomplicated positive cases in the status quo increases the ICER for Scenario 3, which is expected since the status quo becomes a more favorable strategy (Additional file 1: Fig S3). For Scenario 4, decreasing the proportion of uncomplicated positive cases that are treated increases the strategy’s ICER compared to status quo (Additional file 1: Fig S4).

The one-way sensitivity analyses also revealed parameter values that would make Scenarios to be dominated (i.e., less effective and costlier) or dominant (i.e., cost-saving). For example, when the treatment rate of severe negative cases is low, Scenario 4 becomes dominated; on the other hand, when the treatment rate of false positive cases is low (leading to savings), Scenario 4 becomes dominant. For Scenario 5, extremely low treatment rates of test-positive (i.e., true positive) severe causes the strategy to be dominated by the status quo (Additional file 1: Fig S5); for Scenario 6, higher rates of AL treatment in the status quo scenario causes the strategy to be dominated (Additional file 1: Fig S6).

The model found that extremely high values of the RDT testing rate among public providers causes Scenario 2 to be dominated, which means that it leads to less health and higher costs than the status quo (Additional file 1: Fig S2). This may seem like a counterintuitive result, but increasing the RDT testing rate beyond 92%, without any changes in other inputs (e.g., RDT specificity and sensitivity, treatment rate of false negatives), leads to a small decrease in health benefits from individuals receiving a less effective test (i.e., microscopy or clinical diagnosis) in Scenario 2.

To understand the independent effect of malaria prevalence on the cost-effectiveness of Scenarios 1–6 compared to the status quo, additional one-way sensitivity analyses were conducted. Different malaria prevalence levels (0.01 for low, 0.29 for medium, and 0.48 for high) were used in the decision model as indicated in the 2018 MIS [21], and the results are presented in Additional file 1: Table S4. Scenarios 1–6 were found to be less cost-effective in low-prevalence areas like Maputo and more cost-effective in high-prevalence areas like Manica, Zambézia, and Cabo Delgado where malaria prevalence in children has been reported to be between 44 and 57%.

Average costs, health outcomes, and ICERs from the PSA are presented in Table 4. The results in Table 4 represent the average results across 10,000 independent trials where random values of each parameter (Table 2) are used in the model. ICERs from the base case and the PSA differ significantly, implying that parameter uncertainty has a significant effect on the cost-effectiveness of the scenarios modelled. The model also found that except for Scenario 3, all other scenarios had ICERs that were within the cost-effectiveness threshold range ($108–233 in 2019), implying that these interventions may provide economic value on average.

**Table 4:** Average cost-effectiveness results from probabilistic sensitivity analyses

	ICER per life saved	ICER per QALY gained	ICER per DALY averted
Status quo	NA	NA	NA
Scenario 1: 100% testing rate	18 (14–19)	17 (13–22)	13 (12–18)
Scenario 2: Increase testing rate by 10%	13 (10–14)	13 (10–20)	10 (9–13)
Scenario 3: 100% treatment rate	462 (393–754)	640 (626–636)	502 (405–741)
Scenario 4: Increase treatment rate by 10%	81 (75–107)	105 (91–108)	87 (73–100)
Scenario 5: 1 and 3	21 (19–23)	21 (18–26)	30 (25–47)
Scenario 6: 2 and 4	35 (12–56)	36 (9–100)	28 (9–74)

Incremental results are compared to the status quo scenario. 95% credible intervals for the ICERs are in parentheses

DALY, disability-adjusted life year; ICER, incremental cost-effectiveness ratio; NA, not applicable; QALY, quality-adjusted life-year

The cost-effectiveness acceptability curves, which also summarize the results of the PSA, are shown in Additional file 1: Figs S7 and S8. The model found that any cost-effectiveness threshold, Scenario 5 is most likely to be the optimal strategy when QALYs gained was the measure of health benefit used (Additional file 1: Fig S7). On the other hand, when DALYs averted was the measure of health benefit used, Scenario 4 is most likely to be the optimal choice (Additional file 1: Fig S8). The results of the PSA support the base-case results that improvements in treatment, alone or in conjunction with improvements in testing, are more cost-effective than improvements in testing alone.

## Discussion

Using an evidence-based approach to determine funding allocation towards various malaria control strategies, the government of Mozambique aims to increase its capacity to spend resources more effectively. For example, it is taking steps towards improving the economic growth of the country by reaching the planned malaria target of 100% testing and treatment in 2022. The results of this study show that improving testing alone, whether by 10% or reaching the ultimate target of 100%, may not be the most cost-effective strategy. Instead, small or large improvements in treatment, whether alone or in conjunction with improvements in testing, are the most cost-effective strategies for children under five in Mozambique. Findings were sensitive to parameter uncertainty and various model assumptions such as malaria prevalence.

Several malaria cost-effective analyses have been conducted in Mozambique; however, while CEA has been performed on prevention policies, fewer studies have focused on case management. Previous studies in Mozambique evaluated the cost-effectiveness of other artemisinin-based combinations (dihydroartemisinin-piperaquine) as first-line treatment [51] and of a CHW programme including malaria treatment in the services provided [52]. Studies were also conducted in other African countries on home management [53], mass testing and treatment intervention [54], and training shop vendors to perform RDT [55]. By contrast, this study evaluated and compared the costs and health benefits of different case management that can reduce the high burden of malaria in the country.

Important policy implications can be drawn from this study. First, investing resources in improving testing alone is not likely to be the most cost-effective strategy. Alternative solutions should be explored, such as targeting RDTs to high-prevalence areas. Second, under the model’s assumptions, even minor improvements in treatment rates are more cost-effective than alternative scenarios explored, with ICERs that fall within the recommended cost-effectiveness thresholds for Mozambique. Third, the results provide some guidance on the conditions necessary to reduce presumptive treatment or treatment of test-negative cases. Nontreatment of false negatives may lead to premature deaths and morbidity; however, the model found that presumptive treatment of test-negative but febrile cases can be eliminated without reducing overall health benefits provided that test-positive cases are all treated appropriately with ACT. Current treatment guidelines suggest “test-and-treat” (i.e., only providing ACTs to test-positive individuals), which could save resources typically spent on those who are test-negative and do not need treatment; this practice could also limit the development of drug resistance [56]. However, in countries such as Mozambique, where testing is not 100% available, following these guidelines remains difficult. Given current testing availability, findings from this study suggest that nontreatment of *severe* negative cases would always generate a loss in health, while treatment of uncomplicated test-negative cases can be discontinued without any loss in overall health benefits if all test-positive cases are treated. Finally, the model confirms that concurrent improvements in testing and treatment are efficient strategies among children.

This study has several limitations. First, the model developed provides a simplified, high-level representation of malaria care-seeking, testing, and treatment in Mozambique; thus, it necessarily leaves out specific events that occur in practice and may be relevant to decision-makers. For example, this study did not consider comorbidities, though in reality patients with malaria often suffer from other diseases (e.g., HIV infection or malnutrition) that may affect not only how malaria treatment is delivered but also outcomes associated with following treatment. This study also did not model the effect of concurrently implemented malaria prevention strategies. These measures may have a dynamic impact on malaria prevalence, which is a static input in the model (for a complete list of assumptions, see Additional file 1) Second, the CEA model does not consider future related gains from the implementation of testing and treatment strategies. For example, appropriate and timely treatment may reduce the prevalence of malaria and the possibility of community transmission; additionally, if testing improves the appropriate use of ACT, there might be benefits from lower drug resistance that are not incorporated in this analysis [16]. Finally, while the best publicly available data were used to determine the inputs of the model, assumptions needed to be made on some parameters, such as presumptive treatment for severe malaria cases. Parameter uncertainty was addressed through the sensitivity analyses.

## Conclusion

In high malaria burden countries like Mozambique, the impact of malaria interventions can be maximized by identifying and scaling cost-effective strategies for testing and treatment. This study found that improving testing rates alone among children at risk for malaria has the potential to improve health but may not be the most efficient use of limited resources. Instead, small or large improvements in treatment, whether alone or in conjunction with improvements in testing, are the most cost-effective strategies for children under five in Mozambique. Findings from this study were sensitive to parameter uncertainty and various model assumptions, which suggests that additional research to estimate key model inputs can increase the precision of the model’s outputs.

## Supplementary Information

**Additional file 1. ** Supplemental material.

## Data Availability

All data relevant to the study are included in the article or uploaded as additional information. All input parameters used in the decision-analytic model are reported in the main text and additional materials.
