# Supplementary material for: Achieving malaria testing and treatment targets for children under five in Mozambique: a cost-effectiveness analysis
Source: Malar J. 2022 Nov 7;21:320. doi: 10.1186/s12936-022-04354-9 (PMC9641811; doi:10.1186/s12936-022-04354-9)
Supplement: Supplementary file 1 — Additional file 1. Supplemental material. [file 12936_2022_4354_MOESM1_ESM.docx]

**SUPPLEMENTAL MATERIAL**

**Transition Probabilities**

Annual transition probabilities listed in Table 2 in the main text and S1 Table in this Supplementary Material were estimated using peer-reviewed articles, data from the Malaria Indicator Survey (MIS) [2], and gray literature. Several literature searches were conducted by the authors between June-December 2020. We used probabilities specific to children under five where available. Below we detail how each transition probability was estimated.

**S1 Table. Additional model probabilities**

| **Input** | **Base value** | **Range** | **Distribution in PSA** | **Source** |
| --- | --- | --- | --- | --- |
| Severe case given malaria infection | 0.13 | 0.05-0.35 | Beta | [1] |
| Severe cases that seek care | 0.69 | 0.86-0.93 | Beta | [2]^*^ |
| Severe cases that seek care from CHWs given care-seeking | 0.029 | 0.017-0.028 | Beta | [2]^*^ |
| Severe cases that seek care from private providers given care-seeking | 0.0062 | 0.0053-0.0089 | Beta | [2]^*^ |
| Severe cases that are tested | 0.47 | 0.65-0.74 | Beta | [2]^*^ |
| Severe cases that are tested with RDTs by CHWs given testing | 1 | NA | NA | ^†^ |
| Severe cases that are tested with RDTs by private providers given testing | 1 | NA | NA | ^†^ |
| Severe cases that are tested with RDTs by public providers given testing | 1 | NA | NA | ^†^ |
| Severe cases that are treated given a positive test result | 1 | 0.75-1 | Beta | ^†^ |
| Severe cases that are treated given a negative test result | 1 | 0.75-1 | Beta | ^†^ |
| Severe cases that self-treat given no care-seeking | 0.70 | 0.53-0.88 | Beta | [3]^*^ |
| Severe cases that are treated with IV/IM artesunate given a positive result | 0.96 | 0.72-1 | Beta | [4] |
| Severe cases that are treated with ACT or artesunate given a negative result | 0.96 | 0.72-1 | Beta | [4] |
| Severe cases that do not seek care that self-treat with ACT | 0.36 | 0.66-1 | Beta | [2]^*^ |
| Treatment effectiveness of IV/IM artesunate on severe cases | 0.78 | 0.56-1 | Beta | [5] |
| Treatment effectiveness of other drugs on severe cases | 0 | NA | Beta | ^†^ |
| Death after untreated severe malaria | 0.60 | 0.45-0.80 | Beta | [6] |
| Probability of febrile case being non-malaria related | 0.61 | 0.43-0.99 | Beta | [2] |
| Non-cases that seek care | 0.69 | 0.86-0.93 | Beta | [2]^*^ |
| Non-cases that seek care from CHWs given care-seeking | 0.029 | 0.017-0.028 | Beta | [2]^*^ |
| Non-cases that seek care from private providers given care-seeking | 0.0062 | 0.0053-0.0089 | Beta | [2]^*^ |
| Non-cases that are tested | 0.47 | 0.65-0.74 | Beta | [2]^*^ |
| Non-cases that are tested with RDTs by CHWs given testing | 0.69 | 0.52-0.86 | Beta | [7] ^*^ |
| Non-cases that are tested with RDTs by private providers given testing | 0.69 | 0.52-0.86 | Beta | [7]^*^ |
| Non-cases that are tested with RDTs by public providers given testing | 0.69 | 0.52-0.86 | Beta | [7]^*^ |
| Non-cases that are treated given positive test result | 0.99 | 0.74-1 | Beta | [7]^*^ |
| Non-cases that are treated given negative test result | 0.92 | 0.71-1 | Beta | [2]^*^ |
| Non-cases that self-treat given no care-seeking | 0.70 | 0.53-0.88 | Beta | [3]^*^ |
| Non-cases that are treated with AL given a positive result | 0.84 | 0.66-1 | Beta | [2]^*^ |
| Non-cases that are treated with AL given a negative result | 0.05 | 0.66-1 | Beta | [2]^*^ |
| Non-cases that do not seek care that self-treat with AL | 0.36 | 0.66-1 | Beta | [2] ^*^ |
| Accurate clinical diagnosis by CHWs | 0.96 | 0.83-0.99 | Beta | [8, 9]^*^ |
| Accurate clinical diagnosis by private providers | 0.85 | 0.55-0.99 | Beta | [3]^*^ |
| Accurate clinical diagnosis by public providers | 0.76 | 0.54-1 | Beta | [3]^*^ |

^*^Same values as uncomplicated malaria in Table 2 of the main text

^†^Assumed by authors

ACT, artemisinin-based combination therapy; AL, artmether-lumefantrine; CHW, community health worker; IV/IM, intravenous or intramuscular; NA, not applicable; RDT, rapid diagnostic test.

- The malaria prevalence for children (age 6-59 months) is based on the MIS conducted in 2018. The MIS included questionnaires, manuals, and guidelines based on Demographic and Health Surveys materials.[2]
- The distribution for malaria cases by severity is from a retrospective analysis of pediatric cases admitted to a rural hospital in Mozambique. 13.2% of admitted patients had severe malaria indicating 86.8% of malaria cases were uncomplicated.[1]
- Care-seeking behavior among malaria cases and the probability of being tested is based on a cross-sectional study of female household heads in the Zambezia Province in Mozambique. 91% of uncomplicated malaria cases sought care at a health facility, and 67% of those had a rapid diagnostic test (RDT) or a blood smear.[10]
- We also analyzed the 2018 MIS [2] to estimate the probabilities for care-seeking by type of provider and the probability of treatment given positive and negative test results.We used the data to reconstruct the proportions of children under five years old seeking care from private or public providers to get tested or treated; the proportion of those who get tested with RDTs from private or public providers; the proportion of those who test positive for malaria through any test type; the proportion of those who test negative for malaria but still get treated; the proportion of those who seek care from community health workers (CHWs) or private providers when they have severe symptoms; the proportion of those who get tested using RDTs or microscopy when they have malaria symptoms; the proportion of those tested who get treated; the proportion of those who do not seek care, but get treated on their own; the proportion of those with severe malaria who use other treatment than intravenous/intramuscular (IV/IM) artesunate (AS). Since the data do not distinguish between uncomplicated and severe malaria, we assume similar proportions across the two types.
- The probability of being tested with an RDT by type of provider (i.e., CHWs, private providers, and other public providers) is estimated using a cross-sectional study of various health facilities across three provinces (Maputo, Zambezia, and Cabo Delgado) in Mozambique and the results reported in that study are similar to those in other reported studies (68.67%). The probability of testing with microscopy was assumed to complement RDT testing. The data does not distinguish between the type of provider and therefore is assumed to be the same across the three types.[7]
- The sensitivity and specificity of RTDs [11, 12] and microscopy and the accuracy of clinical diagnosis by provider type [3, 8, 9] are based on studies done in and outside Mozambique.
- The probability of treatment with artemether-lumefantrine (AL), artesunate-amodiaquine (AS/AQ), and other drugs following a positive malaria test is based on a secondary analysis of the 2015 Indicators of Immunization, Malaria, and HIV/AIDS Survey.[13] We assumed that the probability of treatment with various drugs among those who received a negative malaria test and those who did not seek care is the same as those with positive test results.
- The effectiveness of AL and AS on uncomplicated and severe malaria, respectively, are based on clinical trials in Mozambique.[14, 5]
- The effectiveness of other drugs on uncomplicated malaria is assumed to be 0 since taking a drug other than an antimalarial provides no benefit.
- Although not Mozambique-specific, literature reports that 70% of patients with suspected uncomplicated malaria in Africa diagnose and treat their illness without proper care.[3]
- For untreated uncomplicated and severe malaria, the risk of death is based on published estimates.[15, 6] Death from other causes than malaria was estimated by subtracting the malaria-related death rate from the all-cause mortality rate for Mozambique reported in the Global burden of Diseases Study.[16]

**Costs**

Cost inputs were based on published literature where available, while some inputs
(e.g., drug costs) were estimated using data from secondary sources. All costs were inflated to 2019 U.S. dollars (US$) using general consumer price index data with 2017 as the reference year. The upper and lower limits of each cost were established by adding and subtracting 25%. Below we detail how each cost was estimated.

- The cost of AL for uncomplicated malaria was determined based on the recommended dosage in the 3^rd^ edition of the World Health Organization (WHO) malaria treatment guidelines [17] and drug costs from the Global Fund to Fight AIDS, Tuberculosis and Malaria’s (The Global Fund) pooled procurement reference price list.[18] The WHO guidelines recommend a dosage of AL twice a day for three days for a total of six doses. The target dose range is 5-24 mg per kg of bodyweight of artemether and 29-144 mg per kg of bodyweight of lumefantrine. For a bodyweight of 5 to 15 kg, a dosage of 20 mg of artemether and 120 mg of lumefantrine is recommended; using costs from the Global Fund ($0.27 per 20/120 mg AL 6x1 tablets), we estimated the total cost of AL for uncomplicated malaria to be $1.62. For a bodyweight of 15-25 kg, 40 mg of artemether and 240 mg of lumefantrine is recommended; using costs from the Global Fund ($0.33 per 80/480 mg 6 tablets), we estimated the total cost of AL to be $1.98. For a bodyweight of 25-35 kg, 60 mg of artemether and 360 mg of lumefantrine is recommended; using costs from the Global Fund ($0.50 per 60/360 mg 6 tablets), we estimated the total cost of AL to be $3.00. For a bodyweight greater than 35 kg, 80 mg of artemether and 480 mg of lumefantrine is recommended; using costs from the Global Fund ($0.63 per 40/240 mg 6 tablets), we estimated the total cost of AL to be $3.78.
- The cost of AS/AQ for uncomplicated malaria was also based on the WHO malaria treatment guideline and Global Fund costs.[17, 18] WHO guidelines recommend a dosage of AS/AQ once a day for three days. The target dose range is 4 (range: 2-10) mg per kg of bodyweight per day of artesunate and 10 (range: 7.5-15) mg per kg of bodyweight per day of amodiaquine. For a bodyweight of 4.5 to 9 kg, a dosage of 25 mg of artesunate and 67.5 mg of amodiaquine is recommended. Using costs from the Global Fund ($0.19 per 25/67.5 mg AS/AQ 3x1), we estimated the cost AS/AQ for uncomplicated malaria to be $0.57. For a bodyweight of 9 to 18 kg, a dosage of 50 mg of artesunate and 135 mg of amodiaquine is recommended. Using costs from the Global Fund ($0.27 per 50/135 mg AS/AQ 3x1), we estimated the cost AS/AQ for uncomplicated malaria to be $0.81. For a bodyweight of 18 to 36 kg, a dosage of 100 mg of artesunate and 270 mg of amodiaquine is recommended. Using costs from the Global Fund ($0.45 per 100/270 mg AS/AQ 3x1), we estimated the cost AS/AQ for uncomplicated malaria to be $1.35. For a bodyweight greater than 36 kg, a dosage of 200 mg of artesunate and 540 mg of amodiaquine is recommended. Using costs from the Global Fund ($0.45 per 100/270 mg AS/AQ 3x1*2 doses), we estimated the cost ASAQ for uncomplicated malaria to be $2.70.
- The cost of other drugs taken by uncomplicated cases was determined based on WHO malaria treatment guidelines [17], which recommend that young children with high fevers are treated with antipyretics such as paracetamol (acetaminophen) at a dose of 15 mg per kg of bodyweight every four hours. For a bodyweight of 5 to 15 kg, 75-225 mg is recommended. The cost of paracetamol was found to be $0.016 for a 500 mg tablet based on generic prices reported in the WHO Model List of Essential Medicines (EML)[19]. Treatment with paracetamol recommendations from the literature suggests contacting a doctor after three days if symptoms persist, indicating six possible doses of paracetamol over three days for a total of 18 doses, for a 1,350-4,050 mg total for 5-15 kg resulting in 2.7-8.1 500 mg tablets. Thus, the cost for 5-15 kg is $.0432-$0.1296 For a bodyweight of 15 to 25kg, a dosage of 225-375 mg is recommended, for a 4,050-6,750 mg total, resulting in 8.1-13.5 500 mg tablets, and the cost of other drugs by uncomplicated cases is $0.1296-$0.216. For a bodyweight of 25-35 kg, a dosage of 375-525 mg is recommended, for a 6,750-9,450 mg, resulting in 13.5-18.9 500 mg tablets, and the cost of other drugs by uncomplicated cases is $0.216-$0.3024. For a bodyweight greater than 35 kg, a dosage of 540 mg is recommended, for a 9,720 mg total, resulting in 19.44 500 mg tablets, and the cost of other drugs by uncomplicated cases is $0.31104.
- The cost of AS for severe malaria was based on the WHO malaria treatment guidelines, which recommend treating children with severe malaria with IV/IM AS for at least 24 hours and complete treatment within three days of artemisinin-based combination therapy (ACT). The guidelines further recommend that children weighing less than 20 kg receive a higher dose of artesunate 3 mg per kg of bodyweight per dose to ensure exposure to the drug. For a bodyweight greater than 20 kg, 2.4 mg/kg dose is recommended. For a bodyweight of 5 to 15 kg, a dosage of 15-45 mg is recommended. Based on this recommended dosage, which costs $1.45 per vial (30 and 60 mg powder for solution for injection) and adding the costs of AL for three days ($1.62), we estimated the total cost of AS for severe malaria 5-15kg to be $3.07. For a bodyweight of 15 to 25 kg, a dosage of 45-60 mg is recommended. Based on this recommended dosage, which costs $1.45 per vial, and adding the costs of AL for three days ($1.98), we estimated the total cost of AS for severe malaria 15-25kg to be $3.43. For a bodyweight of 25 to 35 kg, 60-84 mg is recommended. Based on this recommended dosage, which costs $1.45 per vial (1-2 vials needed) and adding the costs of AL for three days ($3.00), we estimated the total cost of AS for severe malaria 25-35 kg to be $4.45-$5.90. For a bodyweight greater than 35 kg, 84 mg is recommended. Based on this recommended dosage, which costs $1.45 per vial (2 vials needed) and adding the costs of AL for three days ($3.78), we estimated the total cost of AS for severe malaria greater than 35 kg to be $6.68.
- The cost of microscopy is based on a Mozambique study that estimated the unit cost of a blood smear to be $0.90 in 2014. The CPI adjustment from 2014 to 2019 is 0.71. After adjusting for inflation, the estimated cost is $1.27 per case.
- The cost of RDTs is based on a previous cost-effectiveness analysis of malaria interventions at the community level. The unit cost of RDTs in this study was $1 in 2011, and when adjusted for inflation (CPI adjustment 0.65), it is estimated to be $1.54.
- The cost of clinical diagnosis or service delivery for uncomplicated malaria is calculated from a Mozambique-specific study calculating the household economic burden of malaria.[19] The average health system cost was $4.34 per uncomplicated malaria case, including diagnosis, screening fever, and treatment in 2018. When adjusted to 2019 US$, the cost of clinical diagnosis or service delivery for uncomplicated malaria was assumed to be $4.18 per case.
- The cost of clinical diagnosis or service delivery for severe malaria is calculated from a Mozambique study calculating the household economic burden of malaria.[19] The average health system cost was $36.97 per severe malaria case, including diagnosis, screening fever, and treatment in 2018. When adjusted to 2019 US$, the cost of clinical diagnosis or service delivery for uncomplicated malaria was assumed to be $37.64 per case.

**S2 Table. Summary of alternative results (DALYs)**

|  | **HC/payer costs** | **DALYs averted** | **Incremental costs** | **Incremental DALYs averted** | **ICER (cost per DALY averted)** |
| --- | --- | --- | --- | --- | --- |
| **Status quo** | 1.69 | 0.4169 | NA | NA | NA |
| **Scenario 1: 100% testing rate** | 2.06 | 0.4168 | 0.37 | 0.00011 | 3,486 |
| **Scenario 2: Increase testing rate by 10%** | 1.72 | 0.4169 | 0.03 | 0.00001 | 3,659 |
| **Scenario 3: 100% treatment rate** | 2.03 | 0.4166 | 0.34 | 0.00032 | 1,070 |
| **Scenario 4: Increase treatment rate by 10%** | 1.75 | 0.4168 | 0.06 | 0.00018 | 321 |
| **Scenario 5: 1+3** | 2.61 | 0.4165 | 0.92 | 0.00045 | 2,045 |
| **Scenario 6: 2+4** | 1.77 | 0.4167 | 0.09 | 0.00019 | 457 |

**S2 Table caption:** Incremental results are compared to the status quo. Costs are in 2019 US$.

HC, healthcare; ICER, incremental cost-effectiveness ratio; DALY, disability-adjusted life year.

**S3 Table. Summary of alternative results (lives saved)**

|  | **HC/payer costs** | **Lives saved** | **Incremental costs** | **Incremental lives saved** | **ICER (cost per li saved)** |
| --- | --- | --- | --- | --- | --- |
| **Status quo** | 1.69 | 0.9851 | NA | NA | NA |
| **Scenario 1: 100% testing rate** | 2.06 | 0.9852 | 0.37 | 0.0001 | 3,465 |
| **Scenario 2: Increase testing rate by 10%** | 1.72 | 0.9851 | 0.03 | 0.0000 | 3,637 |
| **Scenario 3: 100% treatment rate** | 2.03 | 0.9854 | 0.34 | 0.0003 | 1,064 |
| **Scenario 4: Increase treatment rate by 10%** | 1.75 | 0.9852 | 0.06 | 0.0002 | 319 |
| **Scenario 5: 1+3** | 2.61 | 0.9855 | 0.92 | 0.0005 | 2,032 |
| **Scenario 6: 2+4** | 1.77 | 0.9853 | 0.09 | 0.0002 | 455 |

**S3 Table caption:** Incremental results are compared to the status quo. Costs are in 2019 US$.

HC, healthcare; ICER, incremental cost-effectiveness ratio.

**S4 Table. Cost-effectiveness of various scenarios under different malaria prevalence**

|  | **Low-prevalence setting (0.01)^*^** | | | **Medium-prevalence setting (0.29)**^†^ | | | **High-prevalence setting (0.48)^‡^** | | |
| --- | --- | --- | --- | --- | --- | --- | --- | --- | --- |
|  | HC/payer costs | QALYs gained | ICER (cost per QALY gained) | HC/payer costs | QALYs gained | ICER (cost per QALY gained) | HC/payer costs | QALYs gained | ICER (cost per QALY gained) |
| **Status quo** | 1.28 | 0.992011 | NA | 1.58 | 0.967477 | NA | 1.69 | 0.9587 | NA |
| **Scenario 1: 100% testing rate** | 1.50 | 0.992013 | 79,203 | 1.91 | 0.967555 | 4,222 | 1.78 | 0.950829 | 3,162 |
| **Scenario 2: Increase testing rate by 10%** | 1.30 | 0.992011 | 74,074 | 1.61 | 0.967483 | 4,349 | 2.19 | 0.950958 | 3,363 |
| **Scenario 3: 100% treatment rate** | 1.76 | 0.992019 | 59,320 | 1.96 | 0.967712 | 1,614 | 1.82 | 0.950839 | 798 |
| **Scenario 4: Increase treatment rate by 10%** | 1.33 | 0.992015 | 11,077 | 1.64 | 0.967608 | 423 | 2.10 | 0.951219 | 272 |
| **Scenario 5: 1+3** | 2.31 | 0.992022 | 89,896 | 2.53 | 0.967808 | 2,870 | 1.84 | 0.951046 | 1,640 |
| **Scenario 6: 2+4** | 1.34 | 0.992015 | 12,929 | 1.66 | 0.967615 | 577 | 2.68 | 0.951377 | 402 |

**S4 Table caption:** Malaria prevalence, which are in parentheses in the top row, are based on the 2018 Malaria Indicator Survey [2] and other analyses [20]. Incremental results are compared to the status quo. Costs are in 2019 US$.

^*^Low-prevalence provinces include Maputo (including Maputo City) and Gaza.

^†^Medium-prevalence provinces include Tete, Sofala, and Inhambane.

^‡^High-prevalence provinces include Manica, Zambézia, Nampula, Niassa, and Cabo Delgado.

HC, healthcare; ICER, incremental cost-effectiveness ratio; QALY, quality-adjusted life year.

**S1 Fig. Tornado diagram for Scenario 1**

**S1 Fig caption:** The tornado diagram shows how the ICER of Scenario 1 in terms of cost per QALY gained changes when parameters in the model are varied from their lowest to highest estimated value one by one, while keeping the other parameters constant. Unless otherwise noted, the parameters listed here are inputs to the status quo scenario. The dashed line is the base-case ICER of Scenario 1 ($3,535 per QALY gained) when compared to the status quo.

^*^Extreme parameter values cause the ICER to become negative due to lower incremental costs and lower incremental benefits compared to the status quo.

AL, artemether-lumefantrine; ICER, incremental cost-effectiveness ratio; QALY, quality-adjusted life year; RDT, rapid diagnostic test.

**S2 Fig. Tornado diagram for Scenario 2**

**S2 Fig caption:** The tornado diagram shows how the ICER of Scenario 2 in terms of cost per QALY gained changes when parameters in the model are varied from their lowest to highest estimated value one by one, while keeping the other parameters constant. Unless otherwise noted, the parameters listed here are inputs to the status quo scenario. The dashed line is the base-case ICER of Scenario 2 ($3,710 per QALY gained) when compared to the status quo.

^*^Extreme parameter values cause the ICER to become negative due to lower incremental costs and lower incremental benefits compared to the status quo.

^†^Extreme values of the parameter cause the ICER to become negative due to lower incremental costs and higher incremental benefits than the status quo.

AL, artemether-lumefantrine; ICER, incremental cost-effectiveness ratio; QALY, quality-adjusted life year; RDT, rapid diagnostic test.

**S3 Fig. Tornado diagram for Scenario 3**

**S3 Fig caption:** The tornado diagram shows how the ICER of Scenario 3 in terms of cost per QALY gained changes when parameters in the model are varied from their lowest to highest estimated value one by one, while keeping the other parameters constant. Unless otherwise noted, the parameters listed here are inputs to the status quo scenario. The dashed line is the base-case ICER of Scenario 3 ($1,085 per QALY gained) when compared to the status quo.

AL, artemether-lumefantrine; ICER, incremental cost-effectiveness ratio; QALY, quality-adjusted life year; RDT, rapid diagnostic test.

^†^Extreme values of the parameter cause the ICER to become negative due to lower incremental costs and higher incremental benefits than the status quo.

**S4 Fig. Tornado diagram for Scenario 4**

**S4 Fig caption:** The tornado diagram shows how the ICER of Scenario 4 in terms of cost per QALY gained changes when parameters in the model are varied from their lowest to highest estimated value one by one, while keeping the other parameters constant. Unless otherwise noted, the parameters listed here are inputs to the status quo scenario. The dashed line is the base-case ICER of Scenario 4 ($325 per QALY gained) when compared to the status quo.

AL, artemether-lumefantrine; ICER, incremental cost-effectiveness ratio; QALY, quality-adjusted life year; RDT, rapid diagnostic test.

^*^Extreme parameter values cause the ICER to become negative due to lower incremental costs and lower incremental benefits compared to the status quo.

^†^Extreme values of the parameter cause the ICER to become negative due to lower incremental costs and higher incremental benefits than the status quo.

**S5 Fig. Tornado diagram for Scenario 5**

**S5 Fig caption:** The tornado diagram shows how the ICER of Scenario 5 in terms of cost per QALY gained changes when parameters in the model are varied from their lowest to highest estimated value one by one, while keeping the other parameters constant. Unless otherwise noted, the parameters listed here are inputs to the status quo scenario. The dashed line is the base-case ICER of Scenario 5 ($2,073 per QALY gained) when compared to the status quo.

AL, artemether-lumefantrine; ICER, incremental cost-effectiveness ratio; QALY, quality-adjusted life year; RDT, rapid diagnostic test.

^*^Extreme parameter values cause the ICER to become negative due to lower incremental costs and lower incremental benefits compared to the status quo.

**S6 Fig. Tornado diagram for Scenario 6**

**S6 Fig caption:** The tornado diagram shows how the ICER of Scenario 6 in terms of cost per QALY gained of changes when parameters in the model are varied from their lowest to highest estimated value one by one, while keeping the other parameters constant. Unless otherwise noted, the parameters listed here are inputs to the status quo scenario. The dashed line is the base-case ICER of Scenario 6 ($464 per QALY gained) when compared to the status quo.

AL, artemether-lumefantrine; ICER, incremental cost-effectiveness ratio; QALY, quality-adjusted life year; RDT, rapid diagnostic test.

^*^Extreme parameter values cause the ICER to become negative due to lower incremental costs and lower incremental benefits compared to the status quo.

^†^Extreme values of the parameter cause the ICER to become negative due to lower incremental costs and higher incremental benefits than the status quo.

**S7 Fig. Cost-effectiveness acceptability curve with QALYs gained as measure of benefit**

**S7 Fig caption:** Cost-effectiveness acceptability curves summarize the results of probabilistic sensitivity analyses. The curves plot the probability that each scenario is cost-effective over a range of cost-effectiveness thresholds.

QALY, quality-adjusted life year.

**S8 Fig. Cost-effectiveness acceptability curve with DALYs averted as measure of benefit**

**S8 Fig caption:** Cost-effectiveness acceptability curves summarize the results of probabilistic sensitivity analyses. The curves plot the probability that each scenario is cost-effective over a range of cost-effectiveness thresholds.

QALY, quality-adjusted life year.

**REFERENCES**

1. Bassat Q, Guinovart C, Sigaúque B, Aide P, Sacarlal J, Nhampossa T, et al. Malaria in rural Mozambique. Part II: children admitted to hospital. Malar J. 2008;7: 37. doi:10.1186/1475-2875-7-37
2. Instituto Nacional de Saúde (INS), ICF. Inquérito nacional sobre indicadores de malária em Moçambique 2018. Maputo and Rockville, MD: INS and ICF; 2019.
3. Hume JC, Barnish G, Mangal T, Armázio L, Streat E, Bates I. Household cost of malaria overdiagnosis in rural Mozambique. Malar J. 2008;7: 33. doi:10.1186/1475-2875-7-33
4. Malaria Care. Notes from the field: improving case management of severe malaria in Mozambique. 2017. Available: <https://malariacare.files.wordpress.com/2017/02/mozambique-severe-malaria-sm.pdf>.
5. Dondorp AM, Fanello CI, Hendriksen IC, Gomes E, Seni A, Chhaganlal KD, et al. Artesunate versus quinine in the treatment of severe falciparum malaria in African children (AQUAMAT): an open-label, randomised trial. The Lancet. 2010;376: 1647–1657. doi:10.1016/S0140-6736(10)61924-1
6. Lubell Y, Mills AJ, Whitty CJM, Staedke SG. An economic evaluation of home management of malaria in Uganda: an interactive markov model. Munayco CV, editor. PLoS ONE. 2010;5: e12439. doi:10.1371/journal.pone.0012439
7. Candrinho B, Plucinski MM, Colborn JM, da Silva M, Mathe G, Dimene M, et al. Quality of malaria services offered in public health facilities in three provinces of Mozambique: a cross-sectional study. Malar J. 2019;18: 162. doi:10.1186/s12936-019-2796-9
8. Boyce MR, O’Meara WP. Use of malaria RDTs in various health contexts across sub-Saharan Africa: a systematic review. BMC Public Health. 2017;17: 470. doi:10.1186/s12889-017-4398-1
9. Boyce MR, Menya D, Turner EL, Laktabai J, Prudhomme-O’Meara W. Evaluation of malaria rapid diagnostic test (RDT) use by community health workers: a longitudinal study in western Kenya. Malar J. 2018;17: 206. doi:10.1186/s12936-018-2358-6
10. Carlucci JG, Blevins Peratikos M, Cherry CB, Lopez ML, Green AF, González-Calvo L, et al. Prevalence and determinants of malaria among children in Zambézia Province, Mozambique. Malar J. 2017;16: 108. doi:10.1186/s12936-017-1741-z
11. Harchut K, Standley C, Dobson A, Klaassen B, Rambaud-Althaus C, Althaus F, et al. Over-diagnosis of malaria by microscopy in the Kilombero Valley, Southern Tanzania: an evaluation of the utility and cost-effectiveness of rapid diagnostic tests. Malar J. 2013;12: 159. doi:10.1186/1475-2875-12-159
12. Plucinski MM, Candrinho B, Dimene M, Colborn J, Lu A, Nace D, et al. Assessing Performance of HRP2 Antigen Detection for Malaria Diagnosis in Mozambique. Loeffelholz MJ, editor. J Clin Microbiol. 2019;57: e00875-19, /jcm/57/9/JCM.00875-19.atom. doi:10.1128/JCM.00875-19
13. Cassy A, Saifodine A, Candrinho B, Martins M do R, da Cunha S, Pereira FM, et al. Care-seeking behaviour and treatment practices for malaria in children under 5 years in Mozambique: a secondary analysis of 2011 DHS and 2015 IMASIDA datasets. Malar J. 2019;18: 115. doi:10.1186/s12936-019-2751-9
14. Nhama A, Bassat Q, Enosse S, Nhacolo A, Mutemba R, Carvalho E, et al. In vivo efficacy of artemether-lumefantrine and artesunate-amodiaquine for the treatment of uncomplicated falciparum malaria in children: a multisite, open-label, two-cohort, clinical trial in Mozambique. Malar J. 2014;13: 309. doi:10.1186/1475-2875-13-309
15. Olliaro P. Mortality associated with severe Plasmodium falciparum malaria Increases with age. Clin Infect Dis. 2008;47: 158–160. doi:10.1086/589288
16. Institute of Health Metrics and Evaluation. GBD results tool. In: Global Health Data Exchange [Internet]. 2018 [cited 19 Oct 2018]. Available: <http://ghdx.healthdata.org/gbd-results-tool>
17. World Health Organization. Guidelines for the treatment of malaria. Geneva: World Health Organization; 2015.
18. The Global Fund to Fight AIDS, Tuberculosis and Malaria. Pooled procurement mechanism reference pricing: antimalarial medicines. The Global Fund; 2021. Available: https://www.theglobalfund.org/media/5812/ppm_actreferencepricing_table_en.pdf
19. Alonso S, Chaccour CJ, Elobolobo E, Nacima A, Candrinho B, Saifodine A, et al. The economic burden of malaria on households and the health system in a high transmission district of Mozambique. Malar J. 2019;18: 360. doi:10.1186/s12936-019-2995-4
20. Ejigu BA. Geostatistical analysis and mapping of malaria risk in children of Mozambique. PLoS ONE. 2020; 15(11): e0241680. doi: 10.1371/journal.pone.0241680.
